# Genome-wide transcriptome analysis reveals that a pleiotropic antibiotic regulator, AfsS, modulates nutritional stress response in *Streptomyces coelicolor *A3(2)

**DOI:** 10.1186/1471-2164-9-56

**Published:** 2008-01-29

**Authors:** Wei Lian, Karthik P Jayapal, Salim Charaniya, Sarika Mehra, Frank Glod, Yun-Seung Kyung, David H Sherman, Wei-Shou Hu

**Affiliations:** 1 Department of Chemical Engineering and Materials Science, University of Minnesota, 421 Washington Ave. SE., Minneapolis, MN 55455, USA; 2 Life Sciences Institute, Departments of Medicinal Chemistry, Chemistry, Microbiology & Immunology, University of Michigan, 210 Washtenaw Ave., Ann Arbor, MI 48109, USA

## Abstract

**Background:**

A small "sigma-like" protein, AfsS, pleiotropically regulates antibiotic biosynthesis in *Streptomyces coelicolor*. Overexpression of *afsS *in *S. coelicolor *and certain related species causes antibiotic stimulatory effects in the host organism. Although recent studies have uncovered some of the upstream events activating this gene, the mechanisms through which this signal is relayed downstream leading to the eventual induction of antibiotic pathways remain unclear.

**Results:**

In this study, we employed whole-genome DNA microarrays and quantitative PCRs to examine the transcriptome of an *afsS *disruption mutant that is completely deficient in the production of actinorhodin, a major *S. coelicolor *antibiotic. The production of undecylprodigiosin, another prominent antibiotic, was, however, perturbed only marginally in the mutant. Principal component analysis of temporal gene expression profiles identified two major gene classes each exhibiting a distinct coordinate differential expression pattern. Surprisingly, nearly 70% of the >117 differentially expressed genes were conspicuously associated with nutrient starvation response, particularly those of phosphate, nitrogen and sulfate. Furthermore, expression profiles of some transcriptional regulators including at least two sigma factors were perturbed in the mutant. In almost every case, the effect of *afsS *disruption was not observed until the onset of stationary phase.

**Conclusion:**

Our data suggests a comprehensive role for *S. coelicolor *AfsS as a master regulator of both antibiotic synthesis and nutritional stress response, reminiscent of alternative sigma factors found in several bacteria.

## Background

Streptomycetes are common saprophytic soil bacteria that constitute some of the most proficient producers of naturally occurring therapeutic molecules like antibiotics, immunosuppressants and anti-cancer agents [1]. The regulation of biosynthesis of these compounds has therefore evoked considerable interest among researchers. It is well-known that antibiotic biosynthesis in bacteria is generally elicited as a physiological response to a variety of environmental stimuli including high cell density, nutritional imbalance and/or presence of stress-inducing agents. In streptomycetes, evidence hinting at the interplay between stress signals and antibiotic synthesis have emerged with the findings that mutants of several *Streptomyces coelicolor *sigma factors implicated in stress-response are also perturbed in antibiotics production [2-8]. Growth limitations resulting from nutritional imbalances like nitrogen or phosphate starvation are also known to trigger antibiotic synthesis [9,10]. Yet, despite extensive efforts aimed at elucidating antibiotic regulatory pathways, the exact chain of molecular events leading from sensing of stress or nutritional starvation signal to the eventual activation of antibiotic pathways remains largely obscure.

Years of genetic research, recently aided by the availability of complete genome sequence of *S. coelicolor *[11], has identified several key players involved in the regulation of secondary metabolism in *S. coelicolor*. One such set of genes is the AfsK-AfsR-AfsS system that globally controls antibiotic synthesis under certain conditions (reviewed in [12,13]). When AfsK, one of numerous serine/threonine kinases in *S. coelicolor*, is autophosphorylated, it phosphorylates AfsR. Phosphorylated AfsR has enhanced DNA-binding activity specific to the promoter of *afsS *and positively regulates its transcription. AfsS is then proposed to lead to the onset of antibiotic production in an as yet unknown manner. Recent studies have uncovered some of the upstream events leading to the activation of this signaling pathway. It has now been proposed that the autophosphorylation of AfsK is caused by increased levels of *S*-adenosyl-_L_-methionine, a probable intracellular signaling factor [14], and that this phosphorylation is modulated by another protein, KbpA [15]. In addition, phosphorylation of AfsR by other serine/threonine kinases like PkaG and AfsL have also been demonstrated [16]. An understanding of the signaling cascade downstream of the AfsK-AfsR-AfsS system, however, continues to remain elusive.

In this study we employ DNA microarrays to perform a genome-wide transcriptome profiling of a *S. coelicolor afsS *disruption mutant. Overexpression of *afsS *in *S. coelicolor *and *S. lividans *had previously been shown to significantly enhance actinorhodin synthesis [17,18] while disruption of *afsS *diminished actinorhodin production [19]. We show here that the regulatory implications of AfsS are not limited to modulation of antibiotic synthesis but extend to the control of certain phosphate starvation response, and nitrogen and sulfate metabolism genes as well. This suggests a far more pleiotropic role for AfsS than that theorized by our current understanding and provides an important link between nutritional stress and activation of antibiotic pathways in *S. coelicolor*. Furthermore, clustering and comparative transcriptome analysis of wild-type and this mutant enabled us to gain insights into the biological functions of certain poorly annotated genes.

## Results and Discussion

### Construction of an *afsS* mutant

The gene *afsS *encodes a relatively small 63-amino-acid "sigma-like" protein containing three repeats of a short 12-amino acid segment that are thought to be crucial for its activity [19]. To investigate the physiological role of AfsS in *S. coelicolor *M145, a disruption mutant was constructed by replacing two of these tandem repeats with an apramycin resistance cassette. The deleted segment was 104 bp in length (+26 to +129 from the translational start site) and the resulting strain was designated as YSK4425.

### Growth and antibiotic production kinetics

**Figure 1:**
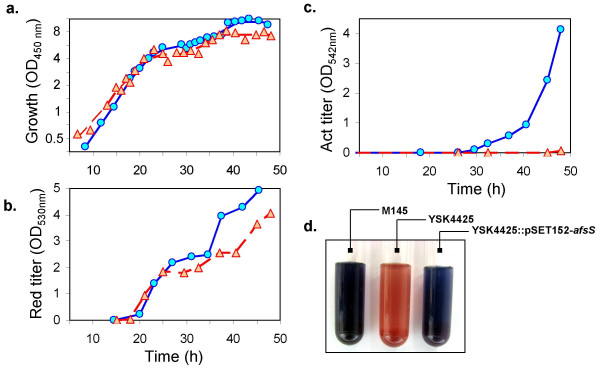
**Growth and antibiotic synthesis kinetics of M145 and YSK4425**. (a) Growth measured using optical density at 450 nm. (b) and (c) Spectrophotometric measurements of antibiotic titers for undecylprodigiosin and actinorhodin. Time profiles for M145 (○, solid blue line) and YSK4425 (△, dashed red line) are shown. The curves represent data from one of two reproducible experiments. (d) Photograph shows the dramatic difference in antibiotic synthesis between the M145 and YSK4425. Pictures are culture samples taken ~4 days after inoculation, indicating that the observed absence of actinorhodin synthesis was a genuine abolishment rather than a delay in synthesis.

Figure 1a–c shows the time profiles of growth and antibiotic titers of M145 and YSK4425 measured during the course of a two-day liquid culture in R5^- ^medium. Disruption of *afsS *did not significantly change the growth kinetics. However, synthesis profiles of two major pigmented antibiotics were altered in the mutant. The most significant change was observed for the polyketide antibiotic, actinorhodin, which normally accumulates to significant levels in M145 giving the culture a distinctive deep blue color. Production of this antibiotic was completely abolished in YSK4425 (Figure 1c and 1d). This finding is much more dramatic than earlier reports where synthesis of actinorhodin was observed in *afsS *deletion strains [19], albeit to a much lesser extent compared to wild-type. This difference was most likely due to our use of R5^- ^medium [20] which does not contain any inorganic phosphate source. Significant actinorhodin accumulation was observed in YSK4425 cultures grown in phosphate containing R2YE medium. Inorganic phosphate depletion is an important trigger for antibiotic synthesis in *S. coelicolor *[21] and, as will be shown later, AfsS is a possible link between phosphate starvation response and actinorhodin synthesis. Another major antibiotic in *S. coelicolor*, a red-pigmented tripyrrole, undecylprodigiosin, accumulated to a slightly lower extent in YSK4425 mainly in stationary phase.

These observations were confirmed by performing an independent biological replicate culture. Genetic complementation of *afsS *in YSK4425 restored antibiotic synthesis (Figure 1d). Taken together, these findings reaffirm the pleiotropic role of *afsS *in regulation of antibiotic synthesis in *S. coelicolor*.

### Microarray analysis

Whole-genome microarrays containing duplicate probes for ~96% of the predicted ORFs in *S. coelicolor *were fabricated as reported earlier [22]. Fluorescently labeled cDNA prepared from total RNA extracts isolated during various growth stages were used in hybridizations. The sampling points chosen for M145 were 15 h, 18 h, 19 h, 21 h, 23 h, 25 h, 27 h, 29 h, 32 h, 34 h, 37 h, 39 h and 42 h; for YSK4425 15 h, 17 h, 19 h, 21 h, 23 h, 25 h, 28 h, 31 h, 32 h, 37 h, 38 h, 41 h and 45 h were chosen. Genomic DNA (gDNA) was used as a universal reference for all hybridizations. The relative fluorescence intensity of cDNA to gDNA, referred to hereafter as the 'log_2 _expression value', is thus an estimate of transcript abundance. Hybridizations were generally performed as duplicates and outlier data points from four spots (duplicate spots from duplicate chips) were filtered out using a mean ± 1.2 times standard deviation cut-off. Key conclusions reported here were confirmed by additional hybridizations performed on the biological replicate culture.

### Identification of kinetically perturbed genes in *afsS* mutant

After normalization, the overall log_2 _expression values for both M145 and YSK4425 samples ranged approximately from -3 to +6. We focused our initial attention on transcripts expressed at moderate to high levels because they show more prominent dynamics allowing higher confidence in identification of differentially expressed genes. Therefore, 2773 genes whose log_2 _expression values rose above zero in at least 10% of all sampled time-points in either strain were considered for initial analysis.

**Figure 2:**
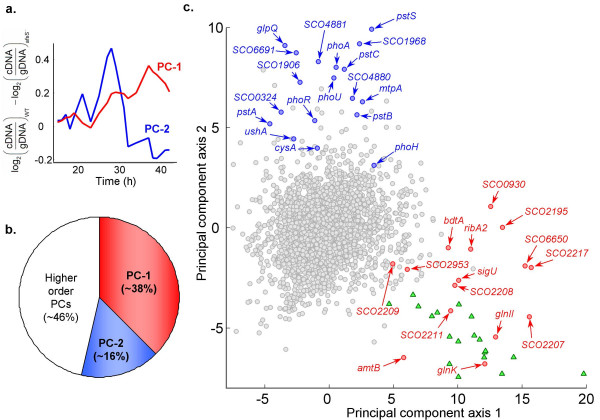
**Identification of patterns among kinetically perturbed genes in YSK4425 using principal component analysis (PCA)**. (a) Eigenvector plots for principal components 1 and 2 – PC-1 (red) and PC-2 (blue) which represent the major patterns in differential expression profiles. PCA was performed on the 'difference profiles' between the two strains to find patterns in differential expression. (b) Fraction of variation accounted for by the first two and the rest of principal components. (c) Plot of PC-1 *vs *PC-2 identifies genes that have a high value along each of PC-1 and PC-2. Actinorhodin biosynthesis genes are shown as filled green triangles, while certain other interesting genes are also marked (red circles for high PC-1 and blue circles for high PC-2). The figure indicates that several functionally related genes, particularly phosphate and nitrogen metabolism related elements, and actinorhodin biosynthesis genes cluster together in various regions of the plot.

To identify kinetically perturbed genes in YSK4425, we first performed a linear interpolation of the time-series data to obtain corresponding values at every time-point in the two series. We then employed two approaches to identify genes with altered profiles in YSK4425. In the first approach, we calculated a 'difference profile' by subtracting log_2 _expression value of genes in YSK4425 from corresponding values in M145. These 'difference profiles' were then analyzed using principal component analysis [see Additional file 1]. Two major patterns (Figure 2a) emerged from this analysis, each displaying a significant deviation from zero at different growth stages. Principal component 1 (PC-1) which accounted for ~38% of all the variance in the 'difference profiles' (Figure 2b) accounted for genes that had progressively higher expression levels in M145 compared to YSK4425 as the culture entered stationary phase. Principal component 2 (PC-2; accounting for ~16% variation) indicated that some genes also had a tendency to display a higher relative expression in M145 between 25 h and 30 h but lower levels thereafter with respect to YSK4425. To identify genes that had extreme values along each of these component axes, we plotted PC-1 versus PC-2 for the selected set moderate/high expression genes (Figure 2c). As we describe in later sections, certain families of genes cluster together in this plot, suggesting coordinate differential regulation for these genes in M145 and YSK4425

In a second complementary approach to identify genes with altered profiles in YSK4425, we calculated Euclidean distance between gene profiles in M145 and YSK4425, normalized by the total number of sampling points [see Additional file 1]. Higher Euclidean distances imply that gene expression patterns in the two strains are dissimilar. In general, many genes with high Euclidean distance – greater than mean (μ) plus 1.2 times the standard deviation (σ) – also had higher component(s) along PC-1 and/or PC-2. These Euclidean distance estimates were used as a means to verify findings from principal component analysis and are reported in relevant tables in later sections.

### Validation of microarray results using qRT-PCR

**Figure 3:**
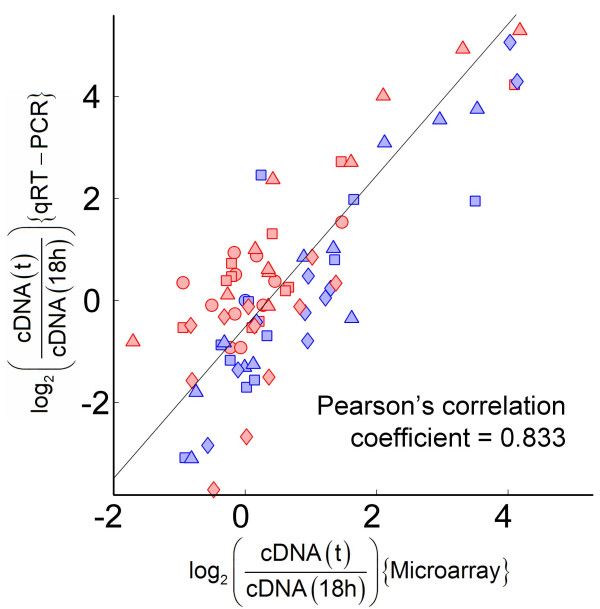
**Validation of microarray results using qRT-PCRs**. A plot of log_2 _expression ratios for 11 different genes (see text) relative to that from the 18 h sample of M145 culture. Samples from 18 h (○), 19 h (□), 21 h (△) and 38 h (◇) for M145 are shown in blue while 17 h (○), 19 h (□), 21 h (△) and 37 h (◇) for YSK4425 are shown in red. A least square straight line fit is also shown.

Quantitative real-time PCRs were performed on reverse transcribed RNA samples to independently validate the microarray results. A total of 11 genes (*actIII*, *redD*, *redP*, *phoR*, *glnII*, *glnK*, *cysD*, *absA1*/*A2*, *scbA *and *scbR*) were chosen for analysis. Relative expression levels from qRT-PCR for samples 18 h, 19 h, 21 h and 38 h in M145 and 17 h, 19 h, 21 h and 37 h in YSK4425 with respect to the 18 h of M145 were plotted against those obtained from microarray experiments (Figure 3). The comparison indicates a good concordance between qRT-PCR and microarray results. We note that the range of dynamics for relative log_2 _ratios obtained from qRT-PCR (-4 to +6) was significantly higher than that from microarray (-2 to +5), indicating that qRT-PCRs are more sensitive particularly in low expression ranges. This probably reflects on the Pearson's correlation coefficient for the plot, resulting in lower value than what could be expected.

### Functional classification of genes with altered expression profiles in *afsS* mutant

Principal component analysis of microarray data not only identified a set of differentially expressed genes in YSK4425, but also enabled their classification into coordinately perturbed subgroups. Each sub-group then represented a distinct pattern of differential expression; the two most commonly observed patterns were briefly mentioned earlier. Further analysis revealed that genes exhibiting each of these patterns also fell within a handful of functional categories (genes marked in Figure 2c) suggesting a role for AfsS in coordinating those functions. These and other related genes are discussed below with reference to Figure 2c.

### Antibiotic biosynthesis genes

**Figure 4:**
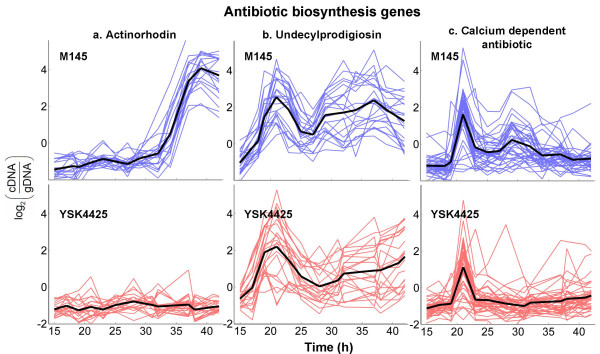
**Time profiles representing 'log_2 _expression value' for antibiotic biosynthesis genes**. Profiles from M145 (blue) and YSK4425 (red) are shown with the average expression profile in each class emphasized by a thick black line.

In R5^- ^medium, *S. coelicolor *M145 begins synthesis of the polyketide antibiotic, actinorhodin, nearly 12 h after entering the stationary phase; antibiotic titers continue to rise in the culture until onset of cell death characterized by gradual fragmentation of mycelia. Microarray analysis revealed that genes belonging to the actinorhodin biosynthesis cluster in M145 were up-regulated slightly ahead of the appearance of blue pigment in culture supernatants. In YSK4425, consistent with the phenotypic observations, we found that these genes were not activated at any stage. By the nature of their profiles in M145 and YSK4425, these genes had a high component along PC-1 discussed earlier (Figure 2c). A direct comparison of the gene expression profiles are shown in Figure 4a. This observation clearly indicates that AfsS exerts its control of actinorhodin synthesis through direct or indirect transcriptional regulation of the relevant biosynthetic genes.

In addition to actinorhodin, at least two other antibiotics, undecylprodigiosin and calcium-dependent antibiotic (CDA) are synthesized by *S. coelicolor*. The expression profiles of both these antibiotic biosynthesis clusters showed a bimodal distribution in M145 with an early peak at 16 h and a later gradual peak between 24 h and 32 h. In YSK4425, while the first peak was present at the same time-point as M145, the second peak for many genes were either absent or delayed (Figures 4b and 4c). These profiles also corroborate with the phenotypic observation that undecylprodigiosin accumulated to a lower extent in YSK4425 compared to M145 particularly in late stationary phase.

### Phosphate starvation response and other similarly regulated genes

**Figure 5:**
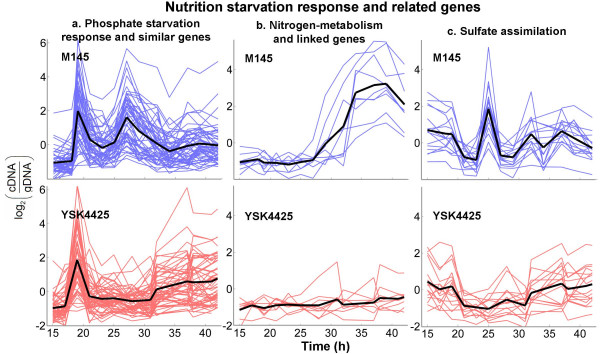
**Time profiles representing 'log_2 _expression value' for nutrient starvation response and related genes**. Profiles from M145 (blue) and YSK4425 (red) are shown with the average expression profile in each class emphasized by a thick black line. The list of genes used in this plot is shown in Table.

Analysis of Figure 2c revealed that many genes with a high value along PC-2 were either annotated as phosphate starvation response elements or were conspicuously related to phosphate metabolism. These genes also displayed distinct bimodal peaks – at around 20 h and 27 h – in M145. A profile search based on correlation coefficient yielded additional genes with similar differential profiles in M145 and YSK4425 (Figure 5a). A total of 56 genes that belonged to this category along with their Euclidean distance values are listed in Table 1. They include genes encoding the two-component system – *phoRP *(*SCO4229–30*), phosphate specific transporters – *pstSCAB*, alkaline phosphatases (*SCO2286*, *phoA *and *SCO0324*) and glycerophosphoryl diester phosphodiesterase (*glpQ*) homologs (*SCO1968 *and *SCO7550*). A large locus (*SCO4873*-*81*) consisting of several hypothetical or membrane proteins as well as those involved in putative phosphorus-free teichuronic acid biosynthesis (*neuAB*) which may substitute for phosphate-rich teichoic acids in cell envelope polymers also belong to this category. A recent study discovered that these genes are PhoP-dependent [23]. In addition, several genes encoding twin-arginine translocation (Tat) dependent exported proteins (*SCO1196*, *SCO1565*, *SCO1633*, *SCO1906*, *SCO2286*, *SCO3790*, *SCO6691 *and *SCO7631*) identified in an earlier study [24] also belonged to this category. Several other genes encoding secreted proteins, nucleases and other hypothetical proteins were also included in this cluster (Table 1). By virtue of their similarity in expression in M145 and differential regulation in YSK4425, we postulate that many of these elements likely belong to the phosphate starvation response system. Transient up-regulation of these genes in M145 might reflect a concerted effort by *S. coelicolor *to counter phosphate starvation by active transport, scavenging, and phosphate regeneration through catabolism from intracellular sources like nucleotides.

**Table 1:** Nutrient metabolism genes with altered expression profiles in YSK4425

**Gene**	**Description**	**Euclidean Distance^†^**
**I. Phosphate starvation response and other similarly regulated genes**		
*SCO0131*	Endonuclease, exonuclease, phosphatase family protein	0.16
*SCO0324*	Putative secreted alkaline phosphatase	0.34
*SCO0546*	Pyruvate carboxylase (*pyc*)	0.13
*SCO0919*	Conserved hypothetical protein	0.15
*SCO0921*	Conserved hypothetical protein	0.10
*SCO1048*	Putative secreted protein	0.32
*SCO1196*	Probable Tat dependent secreted protein	0.36
*SCO1290*	Putative secreted alkaline phosphatase	0.19
*SCO1564*	Putative RNA polymerase sigma factor	0.10
*SCO1565*	Putative glycerophosphoryl diester phosphodiesterase	0.46
*SCO1633*	Tat dependent secreted protein (*tatA*)	0.23
*SCO1906*	Probable Tat dependent secreted protein	0.40
*SCO1968*	Glycerophosphoryl diester phosphodiesterase (*glpQ*)	0.52
*SCO1969*	Putative DNA-methyltransferase	0.20
*SCO2068*	Putative secreted alkaline phosphatase	0.28
*SCO2286*	Putative Tat-dependent secreted alkaline phosphatase (*phoA*)	0.43
*SCO2348*	Putative secreted protein	0.56
*SCO2349*	Hypothetical protein, similar to phosphodiesterase (*phoD*)	0.15
*SCO2532*	PhoH-like protein (*phoH*)	0.18
*SCO2764*	Putative lipoprotein.	0.15
*SCO2878*	Hypothetical protein	0.32
*SCO3789*	Endonuclease, exonuclease, phosphatase family protein	0.10
*SCO3790*	Conserved hypothetical protein	0.35
*SCO4139*	Phosphate ABC transport system ATP-binding protein (*pstB*)	0.32
*SCO4140*	Phosphate ABC transport system permease protein (*pstA*)	0.36
*SCO4141*	phosphate ABC transport system permease protein (*pstC*)	0.41
*SCO4142*	Phosphate-binding protein precursor (*pstS*)	0.57
*SCO4143*	Putative MutT-like protein (*mutT*)	0.09
*SCO4144*	Conserved hypothetical protein	0.20
*SCO4152*	Putative secreted 5'-nucleotidase (*ushA*)	0.27
*SCO4226*	Hypothetical protein	0.30
*SCO4227*	Metallothionein protein (*mtpA*)	0.36
*SCO4228*	Phosphate transport system regulatory protein (*phoU*)	0.37
SCO4229	Sensor kinase (*phoR*)	0.30
SCO4230	Response regulator (*phoP*)	0.19
SCO4401	Putative lipoprotein	0.18
SCO4873	Hypothetical protein	0.25
SCO4877	Hypothetical protein	0.22
SCO4878	Putative glycosyltransferase	0.21
SCO4879	Conserved hypothetical protein	0.28
SCO4880	Putative transferase	0.39
SCO4881	Putative polysaccharide biosynthesis related protein	0.46
SCO5005	Hypothetical protein	0.14
SCO5467	Muramoyl-pentapeptide carboxypeptidase	0.09
SCO5746	Hypothetical protein	0.22
SCO6145	Hypothetical protein	0.34
SCO6691	Putative phospholipase C	0.46
SCO7344	Putative secreted protein	0.16
SCO7550	Putative glycerophosphoryl diester phosphodiesterase	0.29
SCO7631	Putative secreted protein	0.24
SCO7697	Putative secreted hydrolase, phytase (*phy*)	0.30
SCO7722	Conserved hypothetical protein	0.11
**II. Nitrogen utilization and chromosomally linked genes**		
*SCO2207*	Hypothetical secreted protein	0.70
*SCO2208*	Putative caboxylesterase	0.45
*SCO2209*	Putative transcriptional regulator	0.26
*SCO2210*	Glutamine synthetase (*glnII*)	0.63
*SCO2211*	Hypothetical protein	0.45
*SCO2217*	Putative secreted protein	0.69
*SCO2218*	Putative lipoprotein	0.21
*SCO5583*	Ammonium transporter (*amtB*)	0.43
*SCO5584*	Nitrogen regulatory protein PII (*glnK*)	0.59
**III. Sulfate assimilation genes**		
*SCO2910*	Putative cysteine synthase (*cysM*)	0.21
*SCO2911*	Conserved hypothetical protein	0.28
*SCO2912*	Hypothetical protein	0.23
*SCO4164*	Putative thiosulfate sulfurtransferase (*cysA*)	0.43
*SCO4165*	Conserved hypothetical protein	0.32
*SCO4293*	Putative threonine synthase (*thrC*)	0.15
*SCO4294*	Conserved hypothetical protein	0.20
*SCO6094*	Aliphatic sulfonate ABC transporter, permease (*ssuC*)	0.10
*SCO6095*	Aliphatic sulfonate ABC transporter, binding protein (*ssuB*)	0.08
*SCO6096*	Aliphatic sulfonate ABC transporter, binding lipoprotein (*ssuA*)	0.18
*SCO6097*	Sulfate adenylyltransferase subunit 1 (*cysN*)	0.27
*SCO6098*	Sulfate adenylyltransferase subunit 2 (*cysD*)	0.25
*SCO6099*	Adenylylsulfate kinase (*cysC*)	0.19
*SCO6100*	Phosphoadenosine phosphosulfate reductase (*cysH*)	0.21
*SCO6101*	Hypothetical protein	0.21
*SCO6102*	Putative nitrite/sulphite reductase (*cysI*)	0.25

^† ^Euclidean distance normalized with number of time-points. Compare with the average Euclidean distance (*μ*) for all genes = 0.134 and standard deviation (*σ*) = 0.067. Euclidean distances ≥ *μ *+ 1.2 *σ *indicates significant differential expression. However, there may be other genes which have lower Euclidean distances due to low expression levels. These genes were identified by profile similarity search and difference between M145 and YSK4425 were confirmed by visual inspection.

It is interesting to note that disruption of *afsS *led to perturbation of the phosphate starvation response system. Figure 5a indicates that the second of the characteristic bimodal peaks for these genes were either absent or delayed in YSK4425. Earlier studies in *S. coelicolor *and *S. lividans *using deletion mutants of *phoRP *[25,26], *ppk *[25,27,28], and *pstS *[27] largely concluded that disruption of Pho system components caused a premature phosphate-starvation-like situation leading to overproduction of actinorhodin in these strains. One hypothesis put forward was that phosphorylated PhoP represses transcription of *afsS *and inhibits actinorhodin synthesis until a time when, perhaps, a more severe form of phosphate starvation or another independent mechanism sets in to relieve this repression [21]. However, contrary to expectations from this hypothesis, we observed here that a delay or inactivation of phosphate starvation response genes in stationary phase was accompanied by a complete abolishment of actinorhodin synthesis. Moreover, although it has been hypothesized that *afsS *might be under the control of PhoP, our results indicate that presence of *afsS *is required for normal expression of *phoRP *in stationary phase. Therefore, for the previous hypothesis to be valid, it appears that some form of feedback regulation must exist, whereby AfsS and PhoRP can regulate the transcription of each other.

### Nitrogen metabolism genes and their chromosomal neighbors

The enzyme glutamine synthetase (GS) catalyzes the conversion of NH_4_^+ ^to glutamine, a key step in nitrogen assimilation particularly under low nitrogen conditions. Although, single copies of GS-encoding genes are typically found in most bacteria, the genome of *S. coelicolor *encodes five GS-type enzymes, two of which (*glnA*, *SCO2198 *and *glnII*, *SCO2210*) have so far been confirmed to be involved in nitrogen metabolism [29,30]. In addition, several other genes, including an ammonium transporter (*amtB*, *SCO5583*) and a signal transduction protein PII (*glnK*, *SCO5584*) are implicated in nitrogen assimilation [30,31]. Our microarray analysis revealed that, consistent with previous observations [32], *glnA*, the probable housekeeping glutamine synthetase in *S. coelicolor *was constitutively expressed at high levels in both M145 and YSK4425 (data not shown). However, we found that *glnII*, *glnK *and *amtB *clustered together with actinorhodin biosynthesis genes in Figure 2c indicating differential expression in strain YSK4425. Like actinorhodin biosynthesis genes, these were activated during stationary phase in M145 but remained dormant throughout the culture period in YSK4425 (Figure 5b). Interestingly, a PhoP-deletion mutant also perturbed the expression of *glnII*, *glnK *and *amtB *indicating a link between phosphate and nitrogen starvation response [23]; but, in contrast to our observations, these genes were up-regulated in the absence of PhoP in those experiments. The effect of *glnII *expression on antibiotic synthesis is, however, consistent with previous reports. It has been shown previously that a mutation in *rpoB*, the RNA polymerase β chain, leads to a remarkable increase in *glnII *expression as well as the induction of actinorhodin biosynthesis in an otherwise non-producing strain of *S. lividans *[33]. Also, a recent report found that a homolog of *S. coelicolor *GlnR (the transcriptional regulator of *glnA *and *glnII*) in *Amycolatopsis mediterranei*, when cloned into *S. coelicolor*, had pleiotropic effects on host antibiotics production [34]. These findings suggest that nitrogen starvation response is linked to antibiotic synthesis, and our results propose a central role for AfsS in coordinating this response.

In addition to known nitrogen metabolism genes, certain other chromosomally linked genes also exhibited similar differential profiles in M145 and YSK4425 (Table 1). One of them, *SCO2211*, located immediately downstream of and co-transcribed with *glnII *[35], encodes a putative hypothetical protein with a signal peptide sequence. Further downstream, *SCO2217 *and *SCO2218*, encoding putative secreted and membrane-bound proteins respectively as well as three upstream genes (*SCO2207*-*09*), including a MarR-type transcriptional regulator were similarly regulated. Interestingly, mutational and DNA-binding studies of *SCO2208 *and *SCO2209 *[36] concluded that these were not involved in nitrogen metabolism. Although it has been shown that deletion of *SCO2209 *does not affect GS activity in *S. coelicolor*, one cannot exclude the possibility that these genes are controlled by a common regulator. Our data suggest that transcription of all these genes are either directly or indirectly activated by AfsS activity. It is known that GlnRII (*SCO2213*) is the exclusive transcriptional activator of *glnII *while both GlnRII and its homolog, GlnR bind to the promoter of *amtB*-*glnK*-*glnD *operon [32]. It is therefore likely that the presence of AfsS induces their expression or, perhaps, enhances their DNA-binding ability in an as yet unknown way.

### Sulfate assimilation genes

A closer inspection of Figure 2c revealed that a probable thiosulfate sulfurtransferase (*SCO4164*, *cysA*) had a reasonably high component along PC-2. Although the 'difference profile' of this gene was similar to many phosphate starvation induced genes discussed earlier, the absolute profile in each of M145 and YSK4425 did not resemble them. Instead the profile was characterized by a sharp but transient up-regulation of *cysA *by nearly 60-fold at ~25 h in M145; the mutant, however, lacked this peak. A genome-wide similarity search yielded 15 additional sulfate assimilation genes each having the same (although not as drastic) distinct pattern in both M145 and YSK4425 (Figure 5c and Table 1). We note that these profiles could be reproduced in an independent biological replicate culture and that a representative profile from this set was verified using qRT-PCR. Several genes organized as possible operons including cysteine biosynthesis enzymes (*cysM*, *cysA*, *cysK *and *cysIHCDN*) belonged to this category. Certain aliphatic sulfonate assimilation proteins (*SCO6094*-*96*, *ssuABC*) located immediately downstream of *cysIHCDN *also displayed a similar pattern. An earlier study [6] had reported that several of these genes were activated upon osmotic shock and this activation was mediated by a stress response sigma factor, *σ*^*B *^(*SCO0600*). Yet, many other genes reported to be under the control of *σ*^*B *^were not affected in YSK4425. Thus it appears that while *σ*^*B *^and AfsS may each have a broad set of genes under their control, they probably operate cooperatively in a direct or indirect manner to activate a subset of those genes.

### Other differentially regulated genes

In addition to antibiotic and nutrition starvation genes described above, several other gene profiles were altered in YSK4425. However, these genes might not fit into one of the two major patterns discovered using PCA. Hence, Euclidean distances were used as primary indicators of differential expression to identify additional genes perturbed in YSK4425. Also, unlike earlier cases, not all of the identified genes could be grouped together into functional classes. Therefore, in the absence of additional confidence through similar behavior from functionally related genes, we resorted to a stricter statistical test based on significance analysis of time-series data using profiles from biological replicate cultures (see Methods and [37,38]). This analysis provides a *q*-value for every gene, an estimate of false discovery rate when all genes with lower *q*-values are called as differentially expressed. A *q*-value less than 0.25 was taken as an indication of significant differential expression. We note that at this confidence, the *p*-value for expression profiles were less than 0.008. These calculations were performed in addition to the Euclidean distance estimates described earlier. Genes that passed the Euclidean distance cutoff (≥μ + 1.2σ) in both replicate cultures as well as a *q*-value cutoff (≤ 0.25) from the significance analysis test were further inspected manually and reported in Table 2.

**Table 2:** Differentially expressed regulatory genes identified using Euclidean distance and significance analysis test

**Gene**	**Description**	**Euclidean Distance^†^**	q-value*
*SCO0379*	Catalase (*katA*)	0.38	0.011
*SCO0381*	Putative glycosyl transferase	0.20	0.232
*SCO0382*	UDP-glucose/GDP-mannose family dehydrogenase (*ugd*)	0.30	0.0038
*SCO0383*	Hypothetical protein	0.28	0.035
*SCO0736*	Putative secreted protein	0.33	0.022
*SCO0930*	Putative lipoprotein	0.59	0.123
*SCO1627*	Putative ATP-GTP binding protein (*rarD*)	0.39	0.221
*SCO1629*	Conserved hypothetical protein (*rarB*)	0.39	0.116
*SCO1630*	Putative integral membrane protein (*rarA*)	0.35	0.144
*SCO2195*	Hypothetical protein	0.59	0.236
*SCO2196*	Putative integral membrane protein	0.33	0.053
*SCO2953*	Putative anti sigma factor (*rsuA*)	0.31	0.296
*SCO2954*	Putative RNA polymerase sigma factor (*sigU*)	0.46	0.412
*SCO5147*	putative ECF-subfamily sigma factor (*sigE*)	0.31	0.044
*SCO7251*	Conserved hypothetical protein	0.30	0.169
*SCO7252*	Putative regulatory protein	0.37	0.0038

^† ^Euclidean distance normalized with number of time-points. Compare with the average Euclidean distance (*μ*) for all genes = 0.134 and standard deviation (*σ*) = 0.067. Euclidean distances ≥ *μ *+ 1.2 *σ *indicates significant differential expression.

*q-value from significance analysis of time-series data. Values less than 0.25 are statistically significant.

As with most cases discussed earlier, the effect of *afsS *mutation was not visible until mid/late stationary phase for many of these genes. Among them is a major *S. coelicolor *catalase (*SCO0379*, *catA*) which was up-regulated by nearly eight-fold in M145 during stationary phase but displayed a less prominent increase in YSK4425. A few genes downstream, *SCO0382 *and *SCO0383*, which are categorized as secondary metabolism genes (deoxysugar synthase cluster) displayed a similar pattern. In fact, several genes in the deoxysugar synthase/glycosyl transferase cluster (*SCO0381*-*401*) were similarly regulated (data not shown) although many did not meet our statistical criteria due to smaller fold-changes. The stationary phase activation of another set of genes (*SCO1627*-*30*, *rarABCD*) organized as a single operon was also attenuated in YSK4425. These genes have previously been implicated in control of glucose-dependent activation of actinorhodin biosynthesis in *S. coelicolor *[39]. Interestingly, carbon source dependent modulation of *afsS *has been shown earlier to be responsible for activation of antibiotic synthesis in *S. lividans *[40]. Two additional proteins, *SCO2195–96*, encoding a hypothetical and membrane bound protein respectively were also significantly suppressed in YSK4425 during stationary phase.

The "sigma-like" domains of AfsS do not contain any obvious DNA-binding sites. Yet, as described here, a large set of genes with diverse functions appear to be under its control. This prompted us to search for possible transcriptional regulators differentially expressed in YSK4425 as these could be the potential mediators through which AfsS may exert its control on downstream targets. Our analysis indicated that expression profile of a putative ECF-sigma factor (*SCO5147*) was significantly altered in YSK4425 (Table 2). Consistent with this finding, a recent study had identified this gene as a positive regulator of actinorhodin synthesis in *S. coelicolor *[41]. Moreover, it was also found that activation of *SCO5147 *accompanied the up-regulation of *afsS *in a *redZ *over-expressing strain [42]. The expression profiles of another putative regulator, *SCO7252 *and its upstream neighbor, *SCO7251*, were also dramatically altered in YSK4425. Although these genes have not been previously studied in detail, it is known that *SCO7252 *is homologous to a negative regulator of differentiation and antibiotic synthesis, *nsdA*, in *S. coelicolor *[43]. In addition to these regulators, we note that another sigma factor (*SCO2954*, *sigU*) and its cognate anti-sigma factor (*SCO2953*, *rsuA*) were both suppressed in one of the replicate cultures in YSK4425 during stationary phase (transcript levels nearly eight-fold higher in M145 compared to YSK4425); the difference in the other culture, though, was not as drastic. However, this observation might still be interesting because an *rsuA *deletion strain was significantly delayed in actinorhodin synthesis in *S. coelicolor *[3]. We also note that *sigU *as well as *SCO7251 *and *SCO7252 *were recently reported as differentially expressed in a PhoP-deletion strain [23].

## Conclusion

AfsS-like proteins are relatively rare in nature. Thus far, only a handful of related streptomycetes like *S. lividans*, *S. griseus *and *S. noursei *are known to encode such proteins. With no precedent analyses from model organisms like *Escherichia coli *and *Bacillus subtilis *or closer relatives like *Corynebacteria *and *Mycobacteria*, it should, perhaps, come as no surprise that even the basic molecular function of AfsS has not been elucidated yet. In this work, we have demonstrated that AfsS regulates both antibiotic synthesis and nutrition starvation response genes in *S. coelicolor*. Over 117 genes were perturbed in the *afsS *disruption strain. An overwhelming majority of these genes were down-regulated in the mutant strain indicating that AfsS is primarily a direct or indirect positive regulator of transcription for numerous downstream genes. A common thread linking almost all these observations was that the phenotypic and transcriptional effects of the mutation were not evident until the onset of stationary phase. In this aspect, AfsS resembles alterative sigma factors, many of which have been implicated in stress response and stationary phase adaptation in bacteria. In fact, AfsS is annotated as a "sigma-like" protein because it contains certain conserved residues of the domain 3 of sigma factors [44]. This domain, containing three α-helices, mainly interacts with the β-subunit of RNA polymerase and acts as a linker between domains 2 and 4 [4]. Interestingly, ECF sigma factors, 51 of which are found in *S. coelicolor*, lack this domain and instead contain only a short stretch of linker segment. Thus, an intriguing possibility that may, perhaps, be tested in future studies is if AfsS acts cooperatively with one or more of *S. coelicolor *ECF sigma factors.

One possible hypothesis that can be put forth from this work is that the regulation of actinorhodin synthesis by AfsS is mediated by phosphate starvation genes. Thus, in the absence of AfsS, the phosphate starvation mechanism is impaired leading to the abolishment of actinorhodin synthesis. An alternative hypothesis would suggest that AfsS activates actinorhodin synthesis in an as yet unknown manner, but, under the conditions tested, antibiotic biosynthesis requires sequestering of cellular resources through expression of phosphate, sulfate and nitrogen metabolism genes. Thus, abolishment of actinorhodin synthesis by disruption of *afsS *obviates the need for expression of these genes. A third possible hypothesis would be that the activation of nutrient starvation genes and antibiotic biosynthesis genes are two unrelated activities of AfsS. Further work is needed to test these hypotheses. Irrespective of these possibilities and the mode of action of AfsS, we have shown that it is a master regulator of both antibiotic synthesis and nutrition starvation response genes in *S. coelicolor*. The findings reported here should provide some important clues to unraveling the intricate antibiotic regulatory machinery in *S. coelicolor *and related microbes.

## Methods

### Bacterial strains, growth conditions and assays

*S. coelicolor *A3(2) strains M145 and YSK4425 (Δ*afsS::apr*) were cultured in R5 agar [45] for generation of high concentration spore suspensions. For liquid culture, spores were germinated in 2xYT medium [45] and inoculated into R5^- ^liquid medium [20] lacking sucrose at a density of ~10^7 ^spores/ml. Baffled 2 L flasks containing stainless steel coils with 350 ml working volume were used for liquid cultures. Cultures were incubated at 30°C in an orbital shaker at 300 rpm and cell growth was monitored by measuring optical density at 450 nm. Antibiotic assays were generally carried out as described elsewhere [45]. Since the amounts of intracellular actinorhodin produced was much less than the extracellular γ-actinorhodin under the conditions tested, only data for γ-actinorhodin are shown in the results.

### Disruption of *afsS* and complementation

The *afsS *disruption mutant in *S. coelicolor *M145 was constructed by replacing a major segment of the *afsS *coding region with an apramycin resistance cassette (*apr*). This was achieved by first constructing a disruption plasmid by four-way ligation of a host vector backbone (pDHS901, a derivative of pGM160 *E. coli-Streptomyces *shuttle vector) bearing the thiostrepton resistance gene (*tsr*), a 1 kb fragment identical to the left flanking region of the targeted gene, the *apr *cassette and a 1 kb fragment identical to the right flanking region of the targeted gene. The primers used to amplify the target flanking regions were 5'-GCAAGCTTAGGGCTCGCACCGTTCTCAGC-3' and 5'-GCGAATTCGTCCGCGTCCTTCATCTTGTC-3' for the left segment and 5'-GCCTGCAGACCACGATGGACAACCACATG-3' and 5'-GCGGATCCTACACCCTGGACGCGGTCACC-3' for the right segment. This disruption plasmid was propagated in the methylation deficient *E. coli *host ET12567 and subsequently transformed into *S. coelicolor *M145 through protoplast transformation. Plasmid uptake and homologous recombination was selected for by choosing colonies resistant to apramycin. A subsequent screen for thiostrepton sensitive strains yielded cells that underwent double crossover recombination. Southern hybridizations and PCR were used to confirm the disruption of *afsS*.

Mutants were complemented by reintroduction of the wild-type *afsS *allele. For this, pSET152-*kan*, a modified version of pSET152 (with a *S. coelicolor *bacteriophage *φC31 *recombination site *attB *[46]) was first constructed by inserting a pUC-NEO [47] derived kanamycin resistance gene into its *Pac*I site. An 1192 bp PCR amplified DNA segment comprising 500 bp upstream of start codon (to include the promoter), the wild-type *afsS *gene and 500 bp downstream of stop codon (to include any possible transcription terminators) was then cloned into the *EcoR*I-*Xba*I site of pSET152-*kan*. The resulting plasmid was transferred into *S. coelicolor *by conjugation via a non-methylating *E. coli *donor ET12567 containing pUZ8002 [45]. Reintroduction of the wild-type *afsS *gene in the ex-conjugant was confirmed by PCR and subsequent sequencing.

### RNA extraction

Immediately after harvesting culture extracts, a one-fifth volume of an ice-cold stop solution (5% phenol in ethanol) was added to prevent RNA degradation [48]. The sample was quickly centrifuged at 4°C and cell pellets were stored at -80°C until RNA extraction. To extract RNA, the frozen cell pellets were lyophilized using liquid N_2 _and resuspended in buffer RLT (RNeasy Mini Kit, Qiagen Inc., Valencia, CA). Further steps in RNA extraction were carried out in accordance with the manufacturer's instructions. RNA integrity was determined by gel electrophoresis while the quantity and purity was estimated by absorbance at 260 nm and 280 nm.

### Microarray hybridizations

*S. coelicolor *microarray chips reported earlier [22] were used. 10 μg of total RNA and 200 ng genomic DNA (gDNA) were used in all hybridizations. Total RNA was reverse transcribed to cDNA by SuperscriptII (Invitrogen, Carlsbad, CA) with random hexamer primers (IDT, Coralville, IA) with concomitant incorporation of aminoallyl-dUTP (Ambion, Austin, TX). cDNA was then incubated with Alexa 647 (Invitrogen, Carlsbad, CA) for labeling. Fragmented *S. coelicolor *gDNA was chemically labeled using *Label *IT^® ^Cy3 reagents (Mirus Bio Corp., Madison, WI). The labeled cDNA and gDNA were then mixed and co-hybridized to microarray slides in the presence of 50% formamide. After 16 hours of incubation at 50°C, slides were washed and scanned with ScanArray5000 (Perkin Elmer, Wellesley, MA). Details of all protocols are available online [49].

GenePix (Molecular Devices, Union City, CA) was used to process scanned images and obtain raw intensity data for each spot. The median fluorescence intensity from each channel was used to calculate a log_2 _ratio of cDNA/gDNA. A quantile normalization strategy [22] was adopted to normalize data from multiple chips. The resulting log_2 _ratios were both used to compare transcript levels of the same mRNA in different samples as well as to estimate the relative abundance levels of different mRNA in a single sample.

### Quantitative real-time PCRs

Reverse transcription of total RNA was performed on selected samples with 1 μg of total RNA and 1 μg of random hexamer primer (Amersham Biosciences, Piscataway, NJ) using Superscript™ II RNase H^- ^Reverse Transcriptase (Invitrogen). FullVelocity™ SYBR^® ^Green QPCR kit (Stratagene, La Jolla, CA) was used for real-time quantitative PCRs on Mx3005P (Stratagene). Reactions were carried out in triplicate and appropriate controls were included to verify the absence of gDNA contamination in RNA, and primer-dimer formation. A standard threshold of 0.2 was used on the ROX-normalized log(fluorescence) *vs*. cycle number chart (amplification plot) as a cut-off for C_t _(threshold cycle number). C_t _values were normalized with respect to SCO5820 (*hrdB*), the major vegetative sigma factor of *S. coelicolor*. Relative changes in gene expression were quantified using 2^-ΔΔCt ^method [50] with the 18 h sample from M145 being chosen as the control sample.

### Time-series transcript profile analysis

Certain inherent difficulties associated with liquid cultures of *S. coelicolor *like pellet formation make accurate estimation of growth, and therefore alignment of growth stages between strains, using optical density measurements difficult. Hence microarray-derived global gene expression patterns were used to align wild-type and mutant cultures using a time-warp strategy reported earlier [22,51]. This method aligns the time-frames of different cultures by minimizing the global average Euclidean distance of all gene expression profile pairs. The premise behind this manipulation is that the vast majority of genes are not kinetically differentially expressed between the wild-type and mutant and that only a small set of genes exhibit any differential expression. For cultures performed in the current study, time-warp results suggested an approximate linear shift of adding 5 h for *afsS *mutant profiles with respect to the wild-type. This time-aligned data is reported throughout this manuscript.

Euclidean distance estimates and principal component analysis of the resulting time-series data were used to detect differences between expression profiles of genes in wild-type and mutant. For genes that could not be grouped into coordinately perturbed functional classes, a further statistical assessment was performed through significance analysis of time-series data using EDGE [37,38]. This method, which requires replicate time-series experiments, is based on a statistical framework and uses polynomial regression to account for the temporal nature of the data. For each gene, the null hypothesis was tested by comparing the coefficients of the polynomial fits from the two groups (M145 and YSK4425).

### Microarray data availability

Microarray data used in this study has been made available at NCBI – Gene Expression Omnibus [52] in a MIAME-compliant manner: series accession numbers GSE8107 (M145) and GSE8160 (YSK4425).

## Authors' contributions

WL and KPJ carried out liquid culture experiments, printed DNA microarrays, performed transcriptome experiments, overall data analysis, interpretations and manuscript writing. SC performed qRT-PCR verifications and statistical analysis. SM participated in microarray design and assisted in preliminary data analysis. FG helped with microarray construction and liquid culture experiments. YSK created the mutant strain used in this work. DHS and WSH supervised the study, reviewed results and assisted in manuscript preparation. All authors have read and approved the final manuscript.

## Supplementary Material

Additional file 1 Table of PC-1, PC-2, Euclidean distance and q-values. Complete list of PC-1, PC-2, Euclidean distance and q-value estimates for all the genes are provided in one worksheet. The set of genes passing the Euclidean distance and statistical test cut-offs are shown in a separate worksheet. Click here for file

## References

[B1] Challis GL, Hopwood DA (2003). Synergy and contingency as driving forces for the evolution of multiple secondary metabolite production by *Streptomyces *species. Proc Natl Acad Sci USA.

[B2] Cho YH, Lee EJ, Ahn BE, Roe JH (2001). SigB, an RNA polymerase sigma factor required for osmoprotection and proper differentiation of *Streptomyces coelicolor*. Mol Microbiol.

[B3] Gehring AM, Yoo NJ, Losick R (2001). RNA polymerase sigma factor that blocks morphological differentiation by *Streptomyces coelicolor*. J Bacteriol.

[B4] Gruber TM, Gross CA (2003). Multiple sigma subunits and the partitioning of bacterial transcription space. Annu Rev Microbiol.

[B5] Lee EJ, Cho YH, Kim HS, Ahn BE, Roe JH (2004). Regulation of σ^B ^by an anti- and an anti-anti-sigma factor in *Streptomyces coelicolor *in response to osmotic stress. J Bacteriol.

[B6] Lee EJ, Karoonuthaisiri N, Kim HS, Park JH, Cha CJ, Kao CM, Roe JH (2005). A master regulator sigma governs osmotic and oxidative response as well as differentiation via a network of sigma factors in *Streptomyces coelicolor*. Mol Microbiol.

[B7] Paget MS, Chamberlin L, Atrih A, Foster SJ, Buttner MJ (1999). Evidence that the extracytoplasmic function sigma factor σ^E ^is required for normal cell wall structure in *Streptomyces coelicolor *A3(2). J Bacteriol.

[B8] Sevcikova B, Kormanec J (2004). Differential production of two antibiotics of *Streptomyces coelicolor *A3(2), actinorhodin and undecylprodigiosin, upon salt stress conditions. Arch Microbiol.

[B9] Bystrykh L, Fernandez-Moreno M, Herrema J, Malpartida F, Hopwood D, Dijkhuizen L (1996). Production of actinorhodin-related "blue pigments" by *Streptomyces coelicolor *A3(2). J Bacteriol.

[B10] Doull JL, Vining LC (1990). Nutritional control of actinorhodin production by *Streptomyces coelicolor *A3(2): suppressive effects of nitrogen and phosphate. Appl Microbiol Biotechnol.

[B11] Bentley SD, Chater KF, Cerdeno-Tarraga AM, Challis GL, Thomson NR, James KD, Harris DE, Quail MA, Kieser H, Harper D (2002). Complete genome sequence of the model actinomycete *Streptomyces coelicolor *A3(2). Nature.

[B12] Horinouchi S (2003). AfsR as an integrator of signals that are sensed by multiple serine/threonine kinases in *Streptomyces coelicolor *A3(2). J Ind Microbiol Biotechnol.

[B13] Umeyama T, Lee PC, Horinouchi S (2002). Protein serine/threonine kinases in signal transduction for secondary metabolism and morphogenesis in *Streptomyces*. Appl Microbiol Biotechnol.

[B14] Lee Y, Kim K, Suh JW, Rhee S, Lim Y (2007). Binding study of AfsK, a Ser/Thr kinase from *Streptomyces coelicolor *A3(2) and *S*-adenosyl-L-methionine. FEMS Microbiol Lett.

[B15] Umeyama T, Horinouchi S (2001). Autophosphorylation of a bacterial serine/threonine kinase, AfsK, is inhibited by KbpA, an AfsK-binding protein. J Bacteriol.

[B16] Sawai R, Suzuki A, Takano Y, Lee PC, Horinouchi S (2004). Phosphorylation of AfsR by multiple serine/threonine kinases in *Streptomyces coelicolor *A3(2). Gene.

[B17] Vogtli M, Chang PC, Cohen SN (1994). *afsR2*: a previously undetected gene encoding a 63-amino-acid protein that stimulates antibiotic production in *Streptomyces lividans*. Mol Microbiol.

[B18] Floriano B, Bibb M (1996). *afsR *is a pleiotropic but conditionally required regulatory gene for antibiotic production in *Streptomyces coelicolor *A3(2). Mol Microbiol.

[B19] Lee PC, Umeyama T, Horinouchi S (2002). *afsS *is a target of AfsR, a transcriptional factor with ATPase activity that globally controls secondary metabolism in *Streptomyces coelicolor *A3(2). Mol Microbiol.

[B20] Huang J, Lih CJ, Pan KH, Cohen SN (2001). Global analysis of growth phase responsive gene expression and regulation of antibiotic biosynthetic pathways in *Streptomyces coelicolor *using DNA microarrays. Genes Dev.

[B21] Martin JF (2004). Phosphate control of the biosynthesis of antibiotics and other secondary metabolites is mediated by the PhoR-PhoP system: an unfinished story. J Bacteriol.

[B22] Mehra S, Lian W, Jayapal KP, Charaniya SP, Sherman DH, Hu WS (2006). A framework to analyze multiple time series data: a case study with *Streptomyces coelicolor*. J Ind Microbiol Biotechnol.

[B23] Rodriguez-Garcia A, Barreiro C, Santos-Beneit F, Sola-Landa A, Martin JF (2007). Genome-wide transcriptomic and proteomic analysis of the primary response to phosphate limitation in *Streptomyces coelicolor *M145 and in a Δ*phoP *mutant. Proteomics.

[B24] Widdick DA, Dilks K, Chandra G, Bottrill A, Naldrett M, Pohlschroder M, Palmer T (2006). The twin-arginine translocation pathway is a major route of protein export in *Streptomyces coelicolor*. Proc Natl Acad Sci USA.

[B25] Ghorbel S, Kormanec J, Artus A, Virolle MJ (2006). Transcriptional studies and regulatory interactions between the *phoR*-*phoP *operon and the *phoU*, *mtpA*, and *ppk *genes of *Streptomyces lividans *TK24. J Bacteriol.

[B26] Sola-Landa A, Moura RS, Martin JF (2003). The two-component PhoR-PhoP system controls both primary metabolism and secondary metabolite biosynthesis in *Streptomyces lividans*. Proc Natl Acad Sci USA.

[B27] Diaz M, Esteban A, Fernandez-Abalos JM, Santamaria RI (2005). The high-affinity phosphate-binding protein PstS is accumulated under high fructose concentrations and mutation of the corresponding gene affects differentiation in *Streptomyces lividans*. Microbiology.

[B28] Ghorbel S, Smirnov A, Chouayekh H, Sperandio B, Esnault C, Kormanec J, Virolle MJ (2006). Regulation of *ppk *expression and in vivo function of Ppk in *Streptomyces lividans *TK24. J Bacteriol.

[B29] Fink D, Falke D, Wohlleben W, Engels A (1999). Nitrogen metabolism in *Streptomyces coelicolor *A3(2): modification of glutamine synthetase I by an adenylyltransferase. Microbiology.

[B30] Reuther J, Wohlleben W (2007). Nitrogen metabolism in *Streptomyces coelicolor*: transcriptional and post-translational regulation. J Mol Microbiol Biotechnol.

[B31] Hesketh A, Fink D, Gust B, Rexer HU, Scheel B, Chater K, Wohlleben W, Engels A (2002). The GlnD and GlnK homologues of *Streptomyces coelicolor *A3(2) are functionally dissimilar to their nitrogen regulatory system counterparts from enteric bacteria. Mol Microbiol.

[B32] Fink D, Weissschuh N, Reuther J, Wohlleben W, Engels A (2002). Two transcriptional regulators GlnR and GlnRII are involved in regulation of nitrogen metabolism in *Streptomyces coelicolor *A3(2). Mol Microbiol.

[B33] Lai C, Xu J, Tozawa Y, Okamoto-Hosoya Y, Yao X, Ochi K (2002). Genetic and physiological characterization of *rpoB *mutations that activate antibiotic production in *Streptomyces lividans*. Microbiology.

[B34] Yu H, Yao Y, Liu Y, Jiao R, Jiang W, Zhao GP (2007). A complex role of *Amycolatopsis mediterranei *GlnR in nitrogen metabolism and related antibiotics production. Arch Microbiol.

[B35] Charaniya S, Mehra S, Lian W, Jayapal KP, Karypis G, Hu WS (2007). Transcriptome dynamics-based operon prediction and verification in *Streptomyces coelicolor*. Nucleic Acids Res.

[B36] Weisschuh N, Fink D, Vierling S, Bibb MJ, Wohlleben W, Engels A (2000). Transcriptional analysis of the gene for glutamine synthetase II and two upstream genes in *Streptomyces coelicolor *A3(2). Mol Gen Genet.

[B37] Leek JT, Monsen E, Dabney AR, Storey JD (2006). EDGE: extraction and analysis of differential gene expression. Bioinformatics.

[B38] Storey JD, Xiao W, Leek JT, Tompkins RG, Davis RW (2005). Significance analysis of time course microarray experiments. Proc Natl Acad Sci USA.

[B39] Komatsu M, Kuwahara Y, Hiroishi A, Hosono K, Beppu T, Ueda K (2003). Cloning of the conserved regulatory operon by its aerial mycelium-inducing activity in an *amfR *mutant of *Streptomyces griseus*. Gene.

[B40] Kim ES, Hong HJ, Choi CY, Cohen SN (2001). Modulation of actinorhodin biosynthesis in *Streptomyces lividans *by glucose repression of *afsR2 *gene transcription. J Bacteriol.

[B41] Kang SH, Huang J, Lee HN, Hur YA, Cohen SN, Kim ES (2007). Interspecies DNA microarray analysis identifies WblA as a pleiotropic down-regulator of antibiotic biosynthesis in *Streptomyces*. J Bacteriol.

[B42] Huang J, Shi J, Molle V, Sohlberg B, Weaver D, Bibb MJ, Karoonuthaisiri N, Lih CJ, Kao CM, Buttner MJ, Cohen SN (2005). Cross-regulation among disparate antibiotic biosynthetic pathways of *Streptomyces coelicolor*. Mol Microbiol.

[B43] Li W, Ying X, Guo Y, Yu Z, Zhou X, Deng Z, Kieser H, Chater KF, Tao M (2006). Identification of a gene negatively affecting antibiotic production and morphological differentiation in *Streptomyces coelicolor *A3(2). J Bacteriol.

[B44] Kim CY, Park HJ, Kim ES (2006). Functional dissection of sigma-like domain in antibiotic regulatory gene, *afsR2 *in *Streptomyces lividans*. J Microbiol Biotechnol.

[B45] Kieser T, Bibb MJ, Buttner MJ, Chater KF, Hopwood DA (2000). Practical Streptomyces Genetics.

[B46] Bierman M, Logan R, O'Brien K, Seno ET, Rao RN, Schoner BE (1992). Plasmid cloning vectors for the conjugal transfer of DNA from *Escherichia coli *to *Streptomyces spp*. Gene.

[B47] Williamson JM, Inamine E, Wilson KE, Douglas AW, Liesch JM, Albers-Schonberg G (1985). Biosynthesis of the β-lactam antibiotic, thienamycin, by *Streptomyces cattleya*. Journal of Biological Chemistry.

[B48] Bernstein JA, Khodursky AB, Lin PH, Lin-Chao S, Cohen SN (2002). Global analysis of mRNA decay and abundance in *Escherichia coli *at single-gene resolution using two-color fluorescent DNA microarrays. Proc Natl Acad Sci USA.

[B49] *Streptomyces coelicolor *DNA microarray protocols. https://hugroup.cems.umn.edu/Protocols/protocol.htm.

[B50] Livak KJ, Schmittgen TD (2001). Analysis of relative gene expression data using real-time quantitative PCR and the 2^-ΔΔCt ^method. Methods.

[B51] Aach J, Church GM (2001). Aligning gene expression time series with time warping algorithms. Bioinformatics.

[B52] Gene Expression Omnibus. http://www.ncbi.nlm.nih.gov/geo/.

